# Adiponectin and Nitric Oxide Deficiency-Induced Cognitive Impairment in Fatigued Home-Resident in Mature and Older Adults: A Case-Control Study

**DOI:** 10.1155/2022/7480579

**Published:** 2022-05-11

**Authors:** Ahmad H. Alghadir, Sami A. Gabr, Murad Almomani, Fidaa Almomani, Cynthia Tse

**Affiliations:** ^1^Department of Rehabilitation Sciences, College of Applied Medical Sciences, King Saud University, P.O. Box 10219, Riyadh 11433, Saudi Arabia; ^2^ORL-HNS Department, College of Medicine, King Saud University, Riyadh, Saudi Arabia; ^3^Department of Rehabilitation Sciences, Faculty of Applied Medical Sciences, Jordan University of Science and Technology, Irbid, Jordan; ^4^Faculty of Movement and Rehabilitation Sciences, University of Leuven, Leuven, Belgium

## Abstract

**Objective:**

The present study explores the underlying factors of cognitive abilities in relation to the expression of adiponectin and nitric oxide, fatigue, and other cofounder variables such as physical activity, diabetes, and adiposity status in healthy home-resident mature and older adults.

**Background:**

Fatigue has been shown to be correlated with many metabolic and psychiatric conditions, such as cognitive, neurological, musculoskeletal, and hormonal disorders, as well as physical and unhealthy lifestyles.

**Methods:**

A total of 85 home residents aged 50–85 years participated in this case-control study. Mental, fatigue, and pain status were assessed by the cognitive assessment (LOTCA), fatigue questionnaire (CIS20r), and pain score (0–10). VO_2_ max and the prevalidated global physical activity questionnaire were used to estimate physical status. The levels of adiponectin, nitric oxide (NO), and variables related to diabetes, such as blood sugar and glycated hemoglobin (HbA1c %), were assessed using ELISA and spectrophotometric immunoassays.

**Results:**

The participants were classified according to the CIS-fatigue score into two groups: the healthy group (*n* = 40) and the fatigue group (*n* = 45). In fatigued subjects, LOTCA scores as a measure of cognitive performance significantly decreased (65.97 ± 7.17; *P* = 0.01) as compared with healthy subjects (LOTCA scores, 94.2 ± 7.5). The results of cognitive performance domains (LOTCA seven-subset scores) showed a significant decrease in the scores of orientation, visual perception, spatial perception, motor praxis, vasomotor organization, thinking operations, attention, and concentration in older subjects with fatigue compared with healthy subjects. In addition, pain scores significantly increased, and the expression of both nitric oxide (NO) and adiponectin significantly reduced in older adults with fatigue as compared with healthy controls. The decline in cognitive abilities among older adults with fatigue is significantly associated with the CIS-fatigue score, sedentary lifestyle, obesity, pain status, diabetes, and reduction in the levels of nitric oxide (NO), and adiponectin. Moreover, in fatigued cases, the expression of both NO and adiponectin was significantly correlated with CIS-fatigue score, physical activity, obesity, and diabetes, which indicates its availability as diagnostic markers for cognition in mature and older adults with fatigue.

**Conclusion:**

In the present study, the data concluded that cognitive abilities were significantly associated with the lower expression of adiponectin and NO as endothelial vascular markers in association with fatigue among home-resident older adults. In addition, the reduction in cognition was significantly affected by other parameters, such as diabetes, obesity, and unhealthy sedentary life activities. Moreover, the results might recommend the use of cellular adiponectin and NO as diagnostic indicators of cognitive abilities in fatigued mature and older adults. However, more studies on larger sample sizes are required.

## 1. Introduction

Fatigue has been identified as one of the most significant geriatric conditions that can negatively affect family life, work performance, and social relationships among older adults of both genders [1]. The prevalence rates of fatigue comprise about 10–25% of the general population, whereas more than 50% of older ages suffer from fatigue [2, 3]. In most studies, fatigue was shown to increase with advancing age from 18 to 70 years. However, the estimated differences in fatigue rates between older and younger adults still need to be addressed [4–6]. Fatigue was found to be associated with the limited activity, incapacity, and sedentary lifestyles of older adults [6, 7]. Multiple risk factors, such as physical, neurological, hormonal, and psychiatric disorders, stress, and an unhealthy lifestyle, were shown to be associated with fatigue [7–9]. In many studies, fatigue was associated with many human diseases, such as diabetes, rheumatoid arthritis, musculoskeletal, and cognitive disorders [10–14].

Recently, neuropsychological and neurophysiological changes were reported in patients who recovered from COVID-19 [15]. Many studies have reported a significant link between fatigue, physical activities, metabolic alterations, and cognitive performance in older subjects [16–19]. In a recent study, in cases who participated in considerable physical activity, or at least performed daily exercises, this was shown to have a pivotal role in preventing or ameliorating cancer-related fatigue (CRF) during the active- and posttreatment cancer phases [20].

Previous studies have reported that changes in mood, motivation, and cognition abilities collectively coincide with fatigue status [21]. In most cases, several cognition ability tasks, particularly executive functions and repetition trials, have been shown to be influenced by fatigue [22, 23]. Specific abnormalities in these parameters with other mental tasks have exhibited more difficulties in the changing circumstances of everyday life and personal development of internal goals [24, 25]. Thus, it can influence work productivity and academic performance [26, 27]. However, the burden of fatigue stress on cognitive performance and its correlations based on physiological mechanisms remain to be understood among older adults with fatigue.

Previously, it has been reported that fatigue has a multidimensional state with various origins, including physiological changes such as hormonal abnormalities, unhealthy lifestyles such as poor physical activity which leads to obesity, and disrupted sleep patterns [28–32]. In addition, it was reported that the pathophysiology of fatigue entails abnormalities in the neuroendocrine and immune systems, as well as metabolic changes [33, 34]. At older ages, the potential role of adiponectin and nitric oxide (NO) was reported in many pathological disorders such as cognitive decline, fatigue, diabetes, obesity, inflammation, and physical activity status [28–30, 35, 36].

The lack of behavioral effects of fatigue on cognitive abilities, based on their correlation with both cellular adiponectin and nitric oxide (NO), appeals to a question as to whether fatigue induces cognitive abnormalities via physiological changes in vascular markers such as adiponectin and nitric oxide (NO). In this study, an association was proposed between fatigue, cognitive abilities, and the expression of both adiponectin and nitric oxide as vascular markers in fatigued older adults. The present study, therefore, explores the underlying factors of cognitive abilities in relation to the expression of adiponectin and nitric oxide, fatigue, and other cofounder variables such as physical activity, diabetes, and adiposity status in healthy home-resident mature and older adults.

## 2. Material and Methods

### 2.1. Subjects

**Figure 1:**
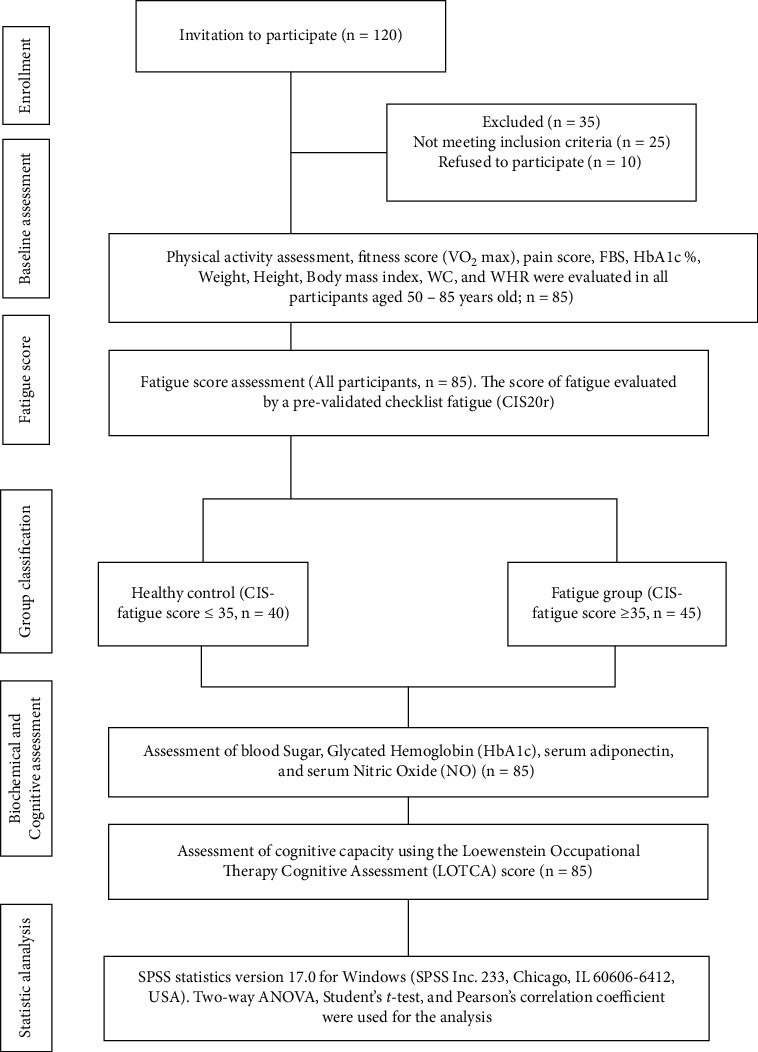
Outline of study procedures.

Using a convenience sampling method, a total of 120 home-resident mature and older adults aged 50–85 years were invited to participate in this case-control study. According to the inclusion criteria, only 85 participants were eligible to participate in this study. Participants who could understand, communicate, and clearly respond to oral instructions; had no severe physical disorders; were clear of endocrine, immune, and psychiatric as well as eating disorders; and were not taking medications such as glucocorticoids, anti-inflammatory, and antioxidant drugs or hormone therapy that might interfere with adiponectin, nitric oxide, and diabetic-related parameters were selected for this study. Based on fatigue scores, the participants were divided into two groups: a healthy group (*n* = 40) and a fatigue group (*n* = 45). All the measurements, such as scores of fatigue (checklist fatigue; CIS20r), cognitive abilities (LOTCA), physical activity (GPAQ; version 2.0), and biochemical parameters, were performed under the supervision of well-trained and qualified physiotherapists and medical staff. In addition, the participants were notified and trained many times before data collection. For each participant, a signed consent form was obtained prior to data collection and biochemical blood profiling.  Figure 1 presents an outline of the research procedure, with all demographic and clinical data reported in Table 1. This study was approved by the Ethical Committee of the Rehabilitation Research Chair of King Saud University (file ID: RRC- 2014-025).

### 2.2. Anthropometric Measurements

Bodyweight (kg) and height (m) were measured with a portable digital metric balance-beam scale and a stationary vertical wall-mounted height board. Body mass and standing height were measured and calibrated to the nearest 0.1 kg and 5 mm, respectively. BMI was subsequently calculated as body mass/height^2^ (kg·m^−2^). The waist circumference (WC) also was measured as the minimum circumference between the iliac crest and the rib cage, and the WHR was calculated as WC divided by the hip measurement.

### 2.3. Assessment of Fatigue Score

Participants' fatigue and pain scores were assessed using a prevalidated checklist fatigue (CIS20r) and pain score (0–10). Test-retest reliability for the checklist fatigue had previously been estimated by using Pearson's R and Spearman's rho statistics. The results showed that CIS20r dimensions had more considerable reliability and validity at a Cronbach's alpha (*α*) value of 0.93 or higher [31, 32]. Four dimensions of fatigue—the subjective experience of fatigue, reduction in motivation, reduction in activity, and reduction in concentration—were measured for each participant within ten minutes [29, 30]. Generally, individuals were classified according to CIS-fatigue scores into healthy subjects (CIS-fatigue score ≤35), heightened fatigue subjects (CIS-fatigue score: 27–35), and subjects with severe fatigue (CIS-fatigue score ≥35) [37, 38]. Thus, based on the CIS-fatigue scores obtained in our study, the participants were classified into only two groups: a fatigue group (CIS-fatigue score ≥35, *n* = 45) and a healthy group (CIS-fatigue score ≤35, *n* = 40).

### 2.4. Assessment of Cognitive Abilities

In recent and previous research studies, LOTCA as a diagnostic tool was successfully used to measure mental health problems among both healthy and older adults with medical complications [39–44]. A prevalidated cognitive assessment (LOTCA), consisting of 7 subsets of questions, was applied to measure cognitive ability among the participants within 30 minutes [39–44]. The test was used for assessing psychiatric domains among participants, where higher scores (123) indicated better cognitive ability and those with minimum scores (27) showed significant degrees of cognitive impairment, as mentioned elsewhere in the literature [39–44]. The participants were classified as normal (LOTCA score: 93–123), moderate (LOTCA score 62–92), and severe cognitive ability (LOTCA score 31–61).

### 2.5. Assessment of Physical Activity

In this evaluation, information on the duration, intensity, and frequency of PA (light, moderate, and vigorous activities) performed in a typical week for each participant was gathered within 10–15 minutes by using a prevalidated global physical activity (PA) questionnaire (GPAQ; version 2.0). In addition, energy expenditure rates were estimated as metabolic equivalent units (METs) derived from PA variables obtained within one week, as defined by the GPAQ. Subsequently, the participants were classified according to energy expenditure into physically inactive (METs-min/week of ≤500, *n* = 25), moderate PA (METs-min/week of 500–2500, *n* = 25), and vigorous PA (≥2500 METs-min/week; *n* = 25) [45–47].

### 2.6. Assessment of Biochemical Parameters

All biochemical parameters were measured in triplicate for all participants.

#### 2.6.1. Analysis of Blood Sugar and Glycated Hemoglobin (HbA1c)

Colorimetric assays were performed to estimate the blood glucose for each subject using glucose oxidase and peroxidase (GOD-POD) kits (Quanti Chrom Glucose Assay Kit, DIGL-100, BioAssay Systems, Hayward, CA, USA). HbA1c was measured using a commercial kit (Bio-Rad, Richmond, CA, USA). The assays were performed according to the instructions provided by the manufacturers. ELISA immune assay kits (Human Insulin ELISA Kit, KAQ1251, Invitrogen Corporation, Camarillo, CA, USA) were used to estimate the levels of insulin in all subjects [47].

#### 2.6.2. Determination of Serum Adiponectin

Adiponectin ELISA kits (ADIPOA; ALPCO Diagnostics, Salem, NH, USA) were used to determine the levels of total adiponectin of all participants. The recovery rate and sensitivity of the test were 0.04 ng/ml and 99%–103% for total adiponectin, respectively. Additionally, the linearity and specificity of the test were estimated in different human serial dilutions, as previously reported in the literature [48].

#### 2.6.3. Determination of Serum Nitric Oxide (NO)

Spectrophotometric analysis was performed to detect the production of NO from the total serum concentrations of stable NO metabolites (nitrites and nitrates) using a Griess reaction. Nitralyser kits (World Precision Instruments Inc.) were used to reduce nitrates to nitrites, and the azo-compound formed due to the Griess reaction was measured at 450 nm. The results were expressed in mmol/l against sodium nitrate as the standard solution [49].

### 2.7. Sample Calculations

Based on the fatigue scores analysis of the participants, a sample comprising 85 subjects was included in this study. The participants were then divided into two groups: a healthy group (*n* = 40) and a fatigue group (*n* = 45). The G ∗ Power program for Windows (version 3.1.9.7) was used to measure the power of the sample size of 85 subjects. Using the *T*-test with a significance level of 0.05, the total sample of 85 achieves a power of 90% with an effect size of 0.64, Df = 83, critical *t* = 1.66, and noncentrality -*α* = 2.95.

### 2.8. Statistical Analysis

All data obtained were analyzed using SPSS statistics version 17.0 for Windows (SPSS Inc. 233, Chicago, IL 60606–6412, USA). Descriptive data were expressed as the mean ± SD. Between subjects, the effects of variables of physical activity levels, pain score, diabetes (insulin, HbA1c (%)), fitness scores (VO_2_ max), obesity (BMI, WHR), and serum biomarkers (NO and adiponectin) on the scores of both fatigue and cognitive abilities were assessed using analysis of covariance (ANCOVA). In addition, Spearman's rank correlation analysis was used to estimate the associations between scores of fatigue and cognitive abilities with NO, adiponectin, and other related parameters of diabetes, obesity, fitness, and physical activity. All the tests were two-tailed, due to the multiple assessments performed. The Bonferroni correction was used to rectify multiple comparisons. The level of significance was established at *P* ≤ 0.05 with 95% CI [50].

## 3. Results

A total of 85 (males = 59, females = 26) home-resident mature and older adults were involved in this study. They were classified according to fatigue scores (CIS scores) into two groups, a fatigue group (CIS-fatigue score 54.35 ± 5.9, *n* = 45) and a healthy group (CIS-fatigue score 11.5 ± 1.81, *n* = 40), as shown in Table (1). For adiposity markers, physical activity, and diabetic status, there was a significant change in waist-to-hip ratio (WHR) (*P* = 0.001), BMI (*P* = 0.01), VO_2_ max; ml/kg ∗ min (*P* = 0.01), physical activity (*P* = 0.01), fasting blood sugar (*P* = 0.04), HbA1c (%) (*P* = 0.03), and insulin (*P* = 0.047) between fatigued and healthy subjects (Table 1).

The cognitive abilities of all participants were measured using LOTCA scores. Compared with healthy subjects (LOTCA scores; 94.2 ± 7.5), subjects with fatigue showed significantly (*P* = 0.01) lower values of LOTCA scores (65.97 ± 7.17) and exhibited a reduction in cognitive performance, leading to a case of cognitive impairment.

**Figure 2:**
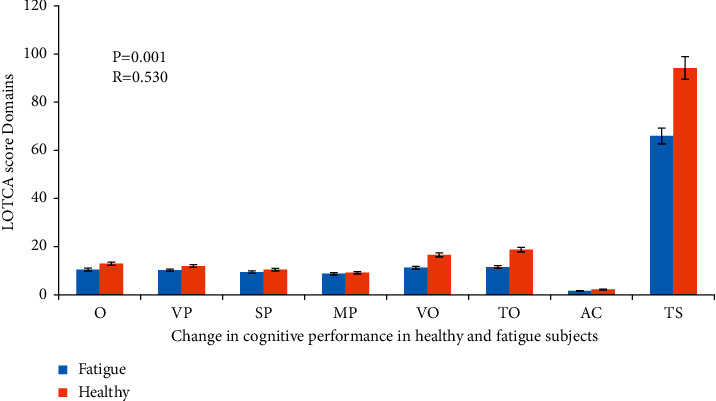
Differences in cognitive performance score domains (LOTCA-7-subset scores) in healthy and fatigued mature and older subjects. Data expressed as means ± SD. There was a significant reduction in the values of cognitive scores (LOTCA-7-subset scores) in cases with fatigue compared to normal healthy controls. For all estimated LOTCA-7-subset scores, cognitive decline was significant at *R* = 0.530 and *P* = 0.001 (fatigue versus control cases), respectively. O: orientation; VP: visual perception; SP: spatial perception; MP: motor praxis; VO: vasomotor organization; TO: thinking operations; AC: attention and concentration; TS: total LOTCA score.

Moreover, in subjects with fatigue, the scores of the domains (LOTCA 7-subset scores) of cognitive performance were significantly reduced (*R* = 0.530, *P* = 0.001) when compared with those of healthy subjects. There were significant decreases in the measured scores of orientation, visual perception, spatial perception, motor praxis, vasomotor organization, thinking operations, attention, and concentration in mature and older subjects with fatigue compared with healthy subjects (Figure 2). Additionally, fatigue and pain scores were shown to be higher (*P* = 0.01) in subjects with fatigue syndromes as compared with healthy normal, as reported in Table 1.

**Figure 3:**
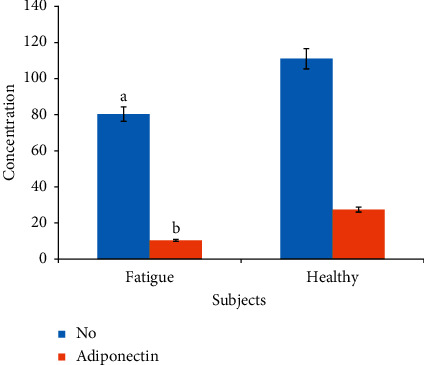
Differences in the levels of nitric oxide (NO) and adiponectin biomarkers markers of healthy and fatigued mature and older adults. Data expressed as means ± SD. In cases with fatigue, the data showed a significant reduction in the levels of both nitric oxide and adiponectin with an estimated *R* = 0.245, ^a^*P* = 0.01 for nitric oxide (NO), and *R* = 0.361, ^b^*P* = 0.01 for adiponectin when compared with normal healthy controls.

The association between the levels of adiponectin and nitric oxide (NO) in subjects with fatigue disorder was studied in all subjects. In participants with fatigue, a significant decrease in the levels of both NO (*R* = 0.245; *P* = 0.01) and adiponectin (*R* = 0.361; *P* = 0.01) was reported compared with healthy adults (Figure 3).

In both groups, fatigue and normal healthy subjects, nitric oxide (NO) and adiponectin were significantly correlated with fatigue, cognitive scores, and other cofounders such as adiposity, diabetic markers, and physical activity. Cognitive and fatigue scores were positively correlated with the decrement in the serum levels of nitric oxide (NO), adiponectin, and the status of pain and negatively with age, BMI, WHR, HbA1c (%), insulin, VO_2_ max, and physical activity (Table 2).

Additionally, an intercorrelation was observed between NO, adiponectin, and other cofounder risk factors. In participants with fatigue, serum levels of NO and adiponectin were positively correlated with physical activity and fitness status and negatively correlated with obesity and markers related to diabetes (Table 3).

## 4. Discussions

In older adults, many health implications, such as physical activity limitations and physiological and mental disorders, are associated with the incidence of fatigue worldwide [6, 51–54]. In this study, a total of 85 home-resident mature and older adults were diagnosed: only 52.9% of the participants had severe fatigue, whereas 47.1% of the participants reported a normal healthy pattern.

Participants with severe fatigue showed a significant increase in all fatigue parameters, such as subjective fatigue, decreased activity, motivation, and concentration when compared with healthy controls with normal fatigue scores. Our findings are in accordance with previous studies, which have reported that more than 50% of older ages suffer from fatigue [2, 3]. Danish older adults aged 70 years old were surveyed to evaluate fatigue. In this study, tiredness, as a measure of fatigue, was reported in 49% of men and 53% of women [55]. In another survey study, chronic fatigue was reported only in 8% of the subjects aged 64–75 years [4]. A clinical health survey on older adults (>50 y) with cancer reported significantly higher ranges of fatigue or exhaustion in 18% of subjects without cancer complications and 25% in cancer cases [56].

Confirming our results, an increase in subjective fatigue and reductions in activity, motivation, and concentration among our participants with fatigue were in line with other results which have recently reported abnormalities in the function of the motor system and areas of the dopaminergic system in the basal ganglia, as measured by magnetic resonance imaging (MRI) of the white matter structures of fatigued young elderly subjects of both genders [57]. These, in turn, demonstrate that fatigue greatly affects motivation and reductions in activity among older adults [57].

In this study, several cofounding risk factors were assessed which may affect the burden or the pathogenesis of fatigue among mature and older adults, especially those with sedentary life activity. Participants with higher fatigue scores significantly showed an increase in adiposity markers (BMI and WHR), reductions in both physical activity and fitness scores (VO_2_ max; ml/kg ∗ min), and higher levels of diabetic markers (FBS, insulin, and HbA1c %) compared with healthy controls with normal fatigue scores. In many studies, fatigue was associated with many human diseases, such as diabetes, [12, 13] rheumatoid arthritis, and musculoskeletal disorders, [14] which affect the physical ability and overall fitness scores among older adults. Many physiological risk factors, especially higher plasma glucose levels, insulin resistance, obesity, depression, and myocardial infarction, were shown to be associated with fatigue syndrome in older adult nurses [58–60].

In our recently published study, lower physical activity, higher adiposity markers, and diabetic-related parameters (HbA1c %, insulin) were shown to be significantly correlated with fatigue scores in patients with type 2 diabetes mellitus (T2DM) [39]. The detrimental effects of lower physical activity and VO_2_ max on the fatigue stage may be related to alterations in the production and utilization of adenosine triphosphate (ATP), limitations in oxygen supply, deficits of blood oxygenation, and oxygen-carrying capacity among subjects with advanced ages [61–64].

In the current study, in order to measure fatigue as a cofounder risk factor for cognitive ability among home-resident mature and older adults, the cognitive function scores were measured. Participants with severe fatigue showed significantly lower cognitive performance in comparison with those with normal fatigue patterns. There was a significant decrease in the cognitive performance scores of orientation, visual perception, spatial perception, motor praxis, vasomotor organization, thinking operations, attention, and concentration in older subjects with severe fatigue as compared with healthy subjects. This is consistent with other studies which have reported an association between fatigue and cognitive disorders in older adults [15–17]. Moreover, many previous studies have linked neurocognitive tasks with fatigue among older adults [65, 66]. Similarly, age significantly influences fatigue, cognitive performance, and associated biomarkers, as previously reported; in older subjects, cognitive performance was shown to be correlated with many associated risk factors, especially fatigue [18–24].

In this study, the reduction in cognitive performance in mature and older adults with severe fatigue revealed a negative correlation with lower physical activity, VO_2_ max, BMI, WHR, and diabetic markers (HbA1c %, insulin). Most clinical and epidemiological studies have reported diabetes to be a severe cofounder risk factor for many pathological as well as mental disorders, especially in older people with cognitive dysfunction and/or fatigue [65–67]. The potential effects of diabetes on cognition may be related to vascularity system disorders among older adults, such as reduced cerebral perfusion and atherosclerosis, along with vascular inflammation [68, 69]. These disorders were shown to be associated with many lifestyle factors such as obesity, poor physical status, and smoking [42].

Our recently published study revealed that increasing physical activity among older adults through moderate aerobic training for 24 weeks provides modulations in both the redox and inflammatory status and overall improvements in cognitive functions [70]. It has also been reported that brain tissue atrophy and neurophysiological changes occur with aging, especially hippocampal volume loss, gray matter, and disturbances in hormonal levels such as changes to the acetyl aspartate/creatine ratio, which may be related to changes in cognitive function [71, 72]. Thus, physical activity interventions aimed to prevent hippocampal atrophy and brain neurodegeneration may prevent cognitive decline and other structural changes as well as alterations in brain function, as reported in previous animal and human studies [73–75]. Brain vascular dysfunctions, chronic inflammation, and neurotransmitter deficits are the most important common processes contributing to cognitive impairment, diabetes, and fatigue in older adults [20, 76, 77].

The bioavailability of both nitric oxide (NO) and adiponectin are very important in the proper functioning of the vascular system, and any abnormal changes may contribute to cognitive declines [78–80]. In this study, we evaluated the roles of adiponectin and NO as markers of vascular changes in cognitive mature and older adults with fatigue. Significant decreases in the levels of both NO and adiponectin were reported in fatigued older adults with poor cognitive performance as compared with healthy adults.

Regarding the contribution of NO in cognitive decline, which is associated with severe fatigue, a recent review of studies discussed the advances in new supportive molecular mechanisms linking the loss of endothelial NO with cognitive decline [81]. The data of our study were in accordance with other studies that have reported significant associations between cognitive decline and losses or decreases in the levels of NO in older adults [20, 82]. It was recently reported that the reduced synthesis and action of NO may be the most vital triggering factor in many brain diseases, such as cognitive decline, dementia, and Alzheimer's disease [83].

However, our data were conversely related to those which have reported higher levels of NO in depressed subjects with cognitive decline [84]. The release of NO is controlled by the overproduction of the nitric oxide enzyme isoforms iNOS rather than eNOS, which induce oxidative stress, cell damage, the activation of immune cells, and subsequent memory impairment [85, 86]. Additionally, the change in the expression of iNOS, which induces NO, was reported in subjects with cognitive decline [86–88]. Therefore, protection from cognitive impairments requires downregulation in the expression of iNOS, as well as the upregulation of eNOS expression [89, 90].

In this study, the adiponectin results were consistent with others which have previously reported lower levels of adiponectin in elderly diabetic patients with moderate cognitive impairment (MCI) [91]. Adiponectin is an adipose-tissue-related protein involved in the regulation of many biological processes such as insulin sensitivity, the homeostasis of glucose, fatty acids, and anti-inflammatory action [92]. The protective role of adiponectin against cognitive decline may be related to its neuroprotective activities on its receptors located in the brain [93]. Its low levels have been established in older humans as well as animal models with cognitive disorders [94, 95]. In contrast to our results, higher levels of adiponectin were shown to be associated with neuroimaging and cognitive outcomes among women aged 70 years old, but its ability to predict neurodegeneration and cognitive decline needs further study [96].

In our study, the data showed that the scores of cognitive performance and fatigue were positively correlated with reductions in the serum levels of both nitric oxide (NO) and adiponectin and negatively correlated with reduced or sedentary physical activity, VO_2_ max, obesity, and diabetes (HbA1c (%), insulin). Moreover, an intercorrelation was observed between NO, adiponectin, and other cofounder risk factors. In fatigued participants with cognitive impairment, serum levels of NO and adiponectin were positively correlated with physical activity and fitness status and negatively correlated with obesity and diabetes.

Some abnormalities in the neuroendocrine and immune systems, as well as metabolic changes as pathophysiological markers, have previously been reported in cases of fatigue [25, 26]. Either adiponectin or nitric oxide (NO) was present in association with many pathological disorders such as fatigue, diabetes, obesity, inflammation, physical activity status, and cognitive decline in older subjects [27, 33–36]. In experimental studies, the chronic loss of endothelial NO was associated with defects in spatial learning and memory among late-middle-aged (LMA) mice deficient in endothelial nitric oxide synthase (LMA eNOS) [82, 96]. The data suggested that the lack or absence of eNOS-derived NO levels has an effective role in the pathogenesis of cognitive decline and fatigue [82, 96]. In addition, it was previously reported that adiponectin has a vasoprotective effect and might be recommended as a potential vascular marker for the inhibition of and protection against many metabolic disorders, such as type 2 diabetes, obesity, and atherosclerosis [94–98].

## 5. Limitations

Although the current sample size showed a correlation between the physiological changes in the levels of adiponectin and NO with the scores of cognitive performance and physical activity, future studies with larger sample sizes are recommended to fully investigate the possible mechanisms of adiponectin and NO as vascular markers and their association with the scores (LOTCA 7-subsets) assessing cognitive function and fatigue consequences in older adults with fatigue.

## 6. Conclusion

In the present study, the data concluded that cognitive abilities were significantly associated with the lower expression of adiponectin and NO as endothelial vascular markers in association with fatigue among home-resident mature and older adults. In addition, the reduction in cognition was significantly affected by other parameters such as diabetes, obesity, and unhealthy sedentary life activities. Moreover, the results recommend the use of cellular adiponectin and NO as diagnostic indicators of cognitive abilities in fatigued mature and older adults. However, more studies on larger sample sizes are required.

## Figures and Tables

**Table 1:** General characteristics of subjects.

Parameters	Healthy group (*n* = 40)	Fatigue group (*n* = 45)	*P* value
Male/female	29/11	30/15	—
Age (years)	59.9 ± 4.1	59.17 ± 4.5	0.12
BMI (kg/m^2^)	22.6 ± 3.72	24.3 ± 2.65	0.01
Waist (cm)	80.0 ± 9.4	82.75 ± 10.34	0.14
Hips (cm)	101.3 ± 10.0	94.3 ± 13.18	0.18
WHR	0.8 ± 0.1	0.98 ± 0.19	0.001
Fitness score (VO_2_ max;ml/kg ∗ min)	41.32 ± 9.8	28.7 ± 3.51	0.01
Fasting blood sugar (mg/dL)	94.0 ± 7.6	110.2 ± 13.9	0.04
HbA1c (%)	4.6 ± 0.84	6.12 ± 1.72	0.03
Insulin	12.01 ± 3.64	9.1 ± 1.23	0.047
Mean LOTCA score (SD)	94.2 ± 7.5	65.97 ± 7.17	0.01
Mean CIS-fatigue score (SD)	11.5 ± 1.81	54.35 ± 5.9	0.01
Pain score (0–10)	2.8 ± 1.4	4.6 ± 3.7	0.01
Physical activity (kcal/week)	5.13 ± 5.1	2.64 ± 0.75	0.01

Values are expressed as mean ± SD; WHR: waist to height ratio; BMI: body mass index; HbA1c: glycated hemoglobin; VO_2_ max: oxygen consumption; CIS: checklist fatigue score. Significance at *P* < 0.05.

**Table 2:** Correlation coefficients analysis between the levels of nitric oxide (NO), adiponectin, adiposity, diabetic markers, and physical activity status based on the scores of fatigue and cognitive abilities in normal and fatigued older adults (*n* = 85).

Parameters	Fatigue (*n* = 45)						Health (*n* = 40)					
	CIS-fatigue scores			Cognitive abilities scores			CIS-fatigue scores			Cognitive abilities scores		
	*R*	(95% CI)	*F*	*R*	(95% CI)	*F*	*R*	(95% CI)	*F*	*R*	(95% CI)	*F*
NO (mmol/l)	0.24^*∗*^	96 (89–100)	3.5	0.75^*∗∗*^	88 (74–100)	2.7	0.45^*∗∗*^	92(86–100)	3.4	0.55^*∗∗*^	90 (78–100)	4.5
Adiponectin (ng/ml)	0.31^*∗*^	94 (86–100)	2.7	0.45^*∗∗*^	95 (91–100)	3.4	0.28^*∗∗*^	90 (89–100)	2.5	0.38^*∗∗*^	92 (89–100)	1.9
Physical activity	−0.21^*∗*^	86 (75–100)	3.4	−0.35^*∗∗*^	88 (76–100)	3.7	−0.25^*∗∗*^	92 (84–100)	2.1	−0.45^*∗∗*^	89 (75–100)	3.7
BMI	−0.13^*∗*^	91 (82–100)	1.8	−0.34^*∗∗*^	88(72–100)	3.5	−0.24^*∗∗*^	87 (70–100)	2.3	−0.44^*∗∗*^	86 (75–100)	1.9
WHR	−0.62^*∗*^	93 (88–100)	2.1	−0.95^*∗∗*^	94 (86–100)	2.8	−0.35^*∗∗*^	90(82–100)	3.4	−0.75^*∗∗*^	95 (82–100)	3.1
Age	−0.32^*∗*^	88 (75–100)	3.7	−0.45^*∗∗*^	87(70–100)	3.1	−0.25^*∗∗*^	91(88–100)	1.8	−0.35^*∗∗*^	89(75–100)	2.1
Insulin	−0.37^*∗*^	91 (86–100)	3.9	−0.54^*∗∗*^	93 (85–100)	2.7	−0.24^*∗∗*^	90 (75–100)	1.9	−0.74^*∗∗*^	89 (75–100)	3.4
HbA1c (%)	−0.84^*∗*^	87 (75–100)	2.4	−0.68^*∗∗*^	85 (72–100)	3.4	−0.48^*∗∗*^	91 (85–100)	3.6	−0.38^*∗∗*^	95(75–100)	4.1
VO_2_ max	−0.71^*∗*^	92 (85–100)	1.8	−0.14^*∗∗*^	88 (82–100)	3.0	−0.65^*∗∗*^	92 (87–100)	4.3	−0.135^*∗∗*^	96 (88–100)	4.6
Pain	0.74^*∗*^	85 (76–100)	2.6	0.125^*∗∗*^	90 (87–100)	2.7	0.85^*∗∗*^	78 (65–100)	3.7	0.115^*∗∗*^	92 (81–100)	3.7

Data presented as a coefficient (*R*); ^*∗*^ denotes significance at <0.05; ^*∗∗*^ denotes significance at <0.01, WHR: waist to height ratio; BMI: body mass index; HbA1c: glycated hemoglobin; VO_2_ max: oxygen consumption; CIS: checklist fatigue score.

**Table 3:** Association between nitric oxide (NO), adiponectin, adiposity, diabetic markers, and physical activity in fatigued older adults (*n* = 45).

Parameters	Fatigue (*n* = 45)						Health (*n* = 40)					
	NO (mmol/l)			Adiponectin (ng/ml)			NO (mmol/l)			Adiponectin (ng/ml)		
	*R*	(95% CI)	*F*	*R*	(95% CI)	*F*	*R*	(95% CI)	*F*	*R*	(95% CI)	*F*
BMI	−0.43^*∗*^	91 (88–100)	2.9	−0.62^*∗∗*^	95 (85–100)	3.1	−0.22^*∗∗*^	89 (76–100)	2.5	−0.52^*∗∗*^	91 (88–100)	3.4
WHR	−0.52^*∗*^	85 (77–98)	1.8	−0.75^*∗∗*^	81 (76–100)	2.6	−0.47^*∗∗*^	91 (85–100)	3.1	−0.65^*∗∗*^	94 (85–100)	2.7
Insulin	−0.35^*∗*^	97 (91–100)	3.1	−0.81^*∗∗*^	92 (86–100)	2.9	−0.32^*∗∗*^	89 (76–100)	4.1	−0.88^*∗∗*^	95 (90–100)	3.8
HbA1c (%)	−0.47^*∗*^	94 (94–100)	2.5	−0.38^*∗∗*^	89(76–100)	3.7	−0.35^*∗∗*^	92(86–100)	2.9	−0.46^*∗∗*^	88 (76–100)	3.6
VO_2_ max	0.54^*∗*^	90 (86–100)	3.6	0.245^*∗∗*^	95 (90–100)	4.3	0.65^*∗∗*^	91(86–100)	3.4	0.195^*∗∗*^	89 (78–100)	3.1
Physical activity	0.45^*∗*^	92(86–100)	3.4	0.87^*∗∗*^	94 (89–100)	3.9	0.46^*∗∗*^	90 (85–100)	3.8	0.92^*∗∗*^	91 (87–100)	3.5

Data presented as a coefficient (R); ^*∗*^ denotes significance at <0.05; ^*∗∗*^ denotes significance at <0.01, WHR: waist to height ratio; BMI: body mass index; HbA1c: glycated hemoglobin; VO_2_ max: oxygen consumption; CIS: checklist fatigue score.

## Data Availability

All data generated or analyzed during this study are presented in the manuscript. Contact the corresponding author for access to the data presented in this study.
